# Comparative analysis of the effectiveness of microsoft copilot artificial intelligence chatbot and google search in answering patient inquiries about infertility: evaluating readability, understandability, and actionability

**DOI:** 10.1038/s41443-025-01056-z

**Published:** 2025-04-22

**Authors:** Tuncer Bahçeci, Burak Elmaağaç, Erman Ceyhan

**Affiliations:** 1 Adnan Menderes University, Faculty of Medicine Department of Urology, Aydın, Turkey; 2 Kayseri City Hospital, Urology Clinic, Kayseri, Turkey; 3 Baskent University, Faculty of Medicine Department of Urology, Ankara, Turkey

**Keywords:** Urogenital reproductive disorders, Sexual dysfunction

## Abstract

Failure to achieve spontaneous pregnancy within 12 months despite unprotected intercourse is called infertility. The rapid development of digital health data has led more people to search for healthcare-related topics on the Internet. Many infertile individuals and couples use the Internet as their primary source for information on infertility diagnosis and treatment. However, it is important to assess the readability, understandability, and actionability of the information provided by these sources for patients. There is a gap in the literature addressing this aspect. This study aims to compare the readability, understandability, and actionability of responses generated by Microsoft Copilot (MC), an AI chatbot, and Google Search (GS), an internet search engine, for infertility-related queries. Prospectively a Google Trends analysis was conducted to identify the top 20 queries related to infertility in February, 2024. Then these queries were entered into GS and MC in May 2024. Answers from both platforms were recorded for further analysis. Outputs were assessed using automated readability tools, and readability scores were calculated. Understandability and actionability of answers were evaluated using the Patient Education Materials Assessment Tool for Printable Materials (PEMAT-P) tool. GS was found to have significantly higher Automated Readability Index (ARI) and Flesch-Kincaid Grade Level (FKGL) scores than MC (*p* = 0.044), while no significant differences were observed in the Flesch Reading Ease, Gunning Fog Index, Simplified Measure of Gobbledygook (SMOG), and Coleman-Liau Index scores. Both GS and MC outputs had readability scores above the 8th-grade level, indicating advanced reading levels. According to PEMAT-P, MC outperformed GS in terms of understandability (68.65 ± 11.99 vs. 54.50 ± 15.09, *p* = 0.001) and actionability (29.85 ± 17.8 vs. 1 ± 4.47, *p* = 0.000). MC provides more understandable and actionable responses to infertility related queries, that it might have great potential for patient education.

## Introduction

Failure to achieve spontaneous pregnancy within 12 months despite unprotected intercourse is called infertility [1]. According to the World Health Organization (WHO), approximately 17.5% of the adult population, or roughly 1 in 6 individuals globally, experience infertility [2]. Today, many infertile individuals use the internet as their primary source of information about infertility diagnosis and treatment [3]. Nowadays, patients can utilize internet search engines and artificial intelligence (AI) chatbots to gather health-related information.

Google, as a search engine, presents a variety of websites in its search results based on the user’s search query, intent, and how its algorithms evaluate and rank those sites [4]. Commonly displayed sites include informational websites (encyclopedias, news sites, educational platforms), health blogs, social media, forums, government and university websites. Over the last two decades, the term “Dr. Google” has begun to be used in the literature as Google has become the foremost visit for millions of people searching for medical information [5, 6].

Large Language Models (LLMs) are deep learning-based AI models that analyze natural language to generate text, summarize information, answer questions, and translate between languages. These capabilities make LLMs ideal for powering AI chatbots, enabling them to interact conversationally, understand questions, and provide relevant answers. AI chatbots have shown near-human performance in tasks like answering medical questions, evaluations and providing text summaries [7–12]. Recent studies have focused on the quality and reliability of information provided by AI chatbots in infertility [13–17]. These studies have shown that AI chatbots deliver promising results, though the reliability of their information has not yet been fully confirmed, indicating potential for future research [13–17].

To date, no studies have evaluated the readability, understandability, and actionability of an AI chatbot and an internet search engine responses in the field of infertility. This study aimed to compare Microsoft Copilot (MC) and Google Search (GS) on these metrics and to determine which tool is more beneficial for patients who are seeking answers related to infertility.

## Material and methods

### Study design and ethics

We adhered the Strengthening the Reporting of Observational Studies in Epidemiology (STROBE) guideline to help authors ensure a high-quality presentation of the observational study conducted [18]. Since this observational study was conducted using publicly available online data and no human subjects were included, Adnan Menderes University Ethics Committee for Non-Interventional Clinical Research confirmed that no ethical approval was required. (Project no: 2024/163).

### Data collection

Prospectively a Google Trends analysis was perfomed on February 23, 2024, to identify the most frequently searched queries related to infertility between February 1, 2023, and January 31, 2024, in a total of nine countries (USA, UK, France, Netherlands, Austria, Italy, Belgium, Spain, Portugal). The Google Trends analysis identified a total of 715 queries, ranked in increments of 50 based on their average monthly search frequency. The most frequently searched query was “IVFs”, (as provided by Google Trends analysis), with an average of 500,000 monthly searches. In second place, “infertility” ranked with an average of 50,000 searches per month. Additionally, there were 22 queries in the category with an average of 50,000–500,000 searches. However, most of these queries were related to location, cost, and duplicate questions. For example, third rank query was “fertility clinic near me,” and the fourth rank query was “IVF cost,” having an average of 50,000 searches per month. We excluded queries pertaining to location, duplicated queries, and queries pertaining to cost from the study and used the 20 most frequently asked queries in the study, as shown in Google Trend analysis results in supplementary file 1.

### Evaluation tools and scoring procedures

In the first phase, a researcher (BE) entered the 20 queries identified by trends analysis into the GS and MC in May 2024. The reason for using MC in our study is its role as a sophisticated processing and editing engine that integrates the capabilities of GPT-4 and other large language models (LLMs). It serves as an accessible AI chatbot for patients, offering up-to-date information without requiring membership and free of charge.

GS output is listed the first short summary answer and is listed after the sponsored advertisement content is considered. For some queries, GS also included visual content. MC output texts and plain texts from the GS answer box were edited by BE for evaluation. To ensure that Uniform Resource Locators (URLs) retrieval was not affected by our search history and existing preferences, we deleted all browsing history, cookies, and cache files before the query was undertaken.

### Readability formulas

These printouts were then entered into an automated readability assessment tool by BE and scores were calculated for each readability formula developed for the English language [19]. The Flesch Reading Ease [20], the Flesch Kincaid Grade Level [21], the Gunning Fog Index [22], the Simplified Measure of Gobbledygook (SMOG) [23], the Coleman-Liau Index [24] and the Automated Readability Index (ARI) [25] readability scores were recorded separately for each query.

### Understandability and actionability scores

The Patient Education Materials Assessment Tool for Printable Materials (PEMAT-P) was used to measure how understandable the written materials were by patients and how actionable the information they contained [26]. The PEMAT-P consists of a total of 24 items: 17 items for understandability and 7 items for actionability. Fifteen items are completed on a 2-point agreement scale (Disagree = 0, Agree = 1) and 9 items are completed on a 3-point agreement scale (Disagree = 0, Agree = 1, and Not applicable). The PEMAT is scored by assigning a value between 0–100 to the items. There is no set threshold for PEMAT-P scores. The higher the score, the more understandable or actionable the material is [27, 28].

### Evaluation of outputs by experts

In the second phase of the study, GS (*n* = 20) and MC (*n* = 20) answers obtained by BE were presented to two experienced urologists (TB, EC) who were fluent in English. These two researchers blindly evaluated the responses with the PEMAT-P tool and scored each query for the PEMAT-P sub-analyses “Understandability” and “Actionability”. After scoring, the agreement between the investigators (TB vs EC) was assessed, and scoring differences were revisited to reach a consensus on the final PEMAT-P scores.

### Statistical analysis

Research data were analyzed using IBM SPSS Statistics for Windows, Version 25.0. Armonk, NY: IBM Corp. statistical program. Shapiro-Wilk test was used to assess normality. Normally distributed data (Coleman-Liau Index, Flesch Reading Ease, Pemat-P Understandability) were compared with the Paired Samples t Test, and non-normally distributed data (the Flesch Kincaid Grade level, the Gunning Fog Index, the Simplified Measure of Gobbledygook (SMOG), the Automated Readability Index (ARI) and Pemat-P actionability) were compared with the Wilcoxon test.

Interrater agreement was evaluated with the Intraclass Correlation Coefficient (ICC), widely used in interrater reliability analyses [29]. The ICC value for Google Search’s actionability was found to be 0.88, indicating good reliability. The other ICC values (the actionability of MC, the understandability of MC, and the understandability of GS) were found to be > 0.90, indicating excellent reliability.

## Results

Readability scores of GS output and MC output were listed in Table 1. Readability scores of GS output and MC output were listed in Table 1. GS scored higher (mean or median) in all readability scores except Flesch Reading Ease (higher mean score for MC). Only the ARI index and Flesch Kincaid Grade Level demonstrated statistically significant differences (*p* = 0.044, for both). PEMAT-P understandability and actionability scores of generated responses by GS and MC were shown in Table 2. The mean PEMAT-P understandability score of GS was 54.50 ± 15.09, and the mean PEMAT-P understandability score of MC was 68.65 ± 11.99. MC was superior to GS in terms of understandability (*p* = 0.001). Also, the actionability score of MC was significantly higher than the actionability score of GS (median 40, range (0–60) vs median 0, range (0–20), *p* = 0.000).

**Table 1:** Comparison of readability scores of google search and microsoft copilot.

Readability Formulas	Score	*P* value
Automated Readability Index (ARI)^a^ Median (min-max)		
Google search	10.51 (2.9–33.7)	0.044*
Microsoft Copilot	8.76 (7.1–14.1)	
Flesch Kincaid Level^a^ Median (min-max)		
Google search	12.28 (7.1–29.1)	0.044^*^
Microsoft Copilot	10.65 (9.0–15.2)	
Gunning Fog Index^a^ Median (min-max)		
Google search	14.01 (9.0–32.5)	0.191
Microsoft Copilot	13.32 (10.8–17.4)	
Simplified Measure of Gobbledygook (SMOG) Index^a^ Median (min-max)		
Google search	13.72 (7.7–24.2)	0.117
Microsoft Copilot	12.32 (11.3–15.5)	
Coleman-Liau Index^b^ Mean ± SD		
Google search	13.1 ± 4.6	0.757
Microsoft Copilot	12.8 ± 2.2	
Flesch Reading Ease^b^ Mean ± SD		
Google search	26.7 ± 24.5	0.100
Microsoft Copilot	36.5 ± 11.5	

*Min* minimum, *max* maximum, *SD* standart deviation.

^a^Wilcoxon test.

^b^Paired samples t test.

^*^statistically signifcant findings.

**Table 2:** Top 20 Infertility Queries and PEMAT-P understandability and actionability scores of generated responses by Google Search and Microsoft Copilot.

Queries	PEMAT-P Understandability^a^ (%)		PEMAT-P Actionability^b^ (%)	
	Google Search	Microsoft Copilot	Google Search	Microsoft Copilot
İvfs	62	50	0	0
Infertility	58	83	0	40
Male fertility test	58	85	20	40
Fertility test for women	42	58	0	20
Fertility testing	58	67	0	60
IVF procedure	80	67	0	40
Conception	67	67	0	0
Check fertility	42	69	0	40
Azoospermia	80	73	0	40
In vitro fertilisation	50	92	0	40
Fertility treatment options	50	67	0	0
IVF fertility	80	75	0	40
Increase male fertility	42	67	0	40
Unexplained infertility	58	75	0	17
Improve male fertility	42	42	0	0
Coenzyme q10 for fertility	47	67	0	40
Cause of infertility in male	42	75	0	40
Cause of infertility in females	67	75	0	40
Best male fertility test	25	50	0	40
Azoospermia treatment	40	69	0	20
Mean ± SD	54.50 ± 15.09	68.65 ± 11.99	1.00 ± 1.00	29.85 ± 3.99

*PEMAT-P* the patient education materials assessment tool for printable materials, *SD* standard deviation.

^a^Paired samples t test.

^b^Wilcoxon test.

## Discussion

The rapid development of digital health data has led more people to search for healthcare-related topics on the Internet. Nowadays, internet search engines, AI chatbots, social media, and health blogs are frequently used tools to gather patient information [30–33]. Patients seek information online before and after the diagnosis of urologic conditions, making web-based health data an integral part of the decision-making process [34]. Many infertile individuals and couples use the Internet as their primary source for information on infertility diagnosis and treatment [3]. In this study, we aimed to compare the digital data provided to patients by GS, which is one of the most popular search engines, and MC, which uses LLM and provides AI-supported data, in terms of readability, understandability, and actionability, to determine which would be more beneficial for patients. In this patient-oriented study, we used free query sites that do not require registration and membership and are easily accessible to patients to be more compatible with patient use in daily life.

The most critical issue in the printed or online presentation of materials used for patient education and information is to create a material that informs and does not mislead the patient using understandable and everyday language. In order to ensure this interaction, materials must be readable and understandable. Readability refers to the ease with which a group of sentences can be understood by the reader and is defined as ‘determining the level of reading comprehension that a person should have to understand written materials with systematic formulas’ [35, 36]. Examples of these formulas include the Flesch Reading Ease, the Flesch Kincaid Grade Level, the Gunning Fog Index, the Simplified Measure of Gobbledygook (SMOG), the Coleman-Liau Index and the Automated Readability Index (ARI) [21, 23, 35–37]. These formulas generally give an idea of the text’s difficulty level by measuring the average number of syllables in words and the average number of words in sentences [38]. Many formulas indicate the grade level at which the text can be understood within the US educational system. For example, The Flesch-Kincaid Grade Level or ARI score of 8.0 indicates that the text can be understood by 8th-grade students [21]. Since readability is related to a large number of parameters, reaching a certain grade level is not a guarantee for the comprehension of information in digital platforms. Which of these formulas to choose depends on the target audience, type, and intended use of the text. Averaging readability scores from several formulas can be supported to ensure reliability [35, 39].

Functional literacy is defined as ‘an individual’s ability to read, write and speak, and to calculate and solve problems at levels of proficiency required to function at work and in society [40]. For people with low functional literacy, there are challenges in finding and interpreting online health information. Therefore, the literature recommends that patient education materials be at an 8th-grade reading level or lower [41–43]. Looking at the literature, in a systematic review examining the readability of online patient education materials for total joint arthroplasty published by Karimi et al., the readability scores of arthroplasty patient education materials produced by websites, web searches, and applications that provide orthopedic-related data were found to be well above the recommended levels [44]. In a recent article, Musheyev et al. examined the responses of AI chatbots (ChatGPT, Perplexity, Chat Sonic, and Microsoft Bing AI) to the most searched queries about urological malignancies (prostate, bladder, kidney, and testis). In this study, the Flesch-Kincaid test was used as the reading score, and it was found to be 11.7(5.7–26) for ChatGPT and 11(4.9–36.6) for Microsoft Bing AI, and it was concluded that reading the responses was quite tricky for AI chatbots [45]. In the study published in 2024 comparing the new ChatGPT with the existing Google Search technology in terms of their ability to provide accurate, practical, and up-to-date information about the actions to be taken after missing a dose of the oral contraceptive pill, it was found that the average word count of ChatGPT responses was significantly longer than the Google Search response box and snippets, and the average Flesh-Kincaid score for ChatGPT responses was 13.10 (university level reading skill), while the average score for Google Search responses was 5.93 (middle school level reading skill) [46]. In the other study investigating the quality and accessibility of answers to common transgender health questions, the Flesch-Kincaid Grade Level of ChatGPT’s responses was found to range from 15.0–17.7 [17].

Robins et al. published in 2016, which examined Canadian and international websites on male infertility and male fertility preservation, SMOG, Fog, and Flesch-Kincaid grade levels were examined from Readability index scores, and the scores were found to be 13.92, 14.59, and 12.89, respectively. This study found that automated readability indices of online information on male infertility and male fertility preservation had very low readability overall and were particularly relevant to those with post-secondary education [47]. A study evaluating the readability of websites in previous years found that many websites had reading levels suitable for individuals with at least a high school education [48]. In 2023, in a study evaluating educational material on websites about dysphagia, readability scores (FRE 46.34 ± 13.59, F-KGL 10.26 ± 2.29, FOG 12.11 ± 2.08, and SMOG 12.38 ± 1.70) showed that the average reading level corresponded to grade 11 [27]. In our study, both GS and MC outputs responded at a readability level above grade 8, which is well above the levels recommended in the literature for readability. Similar to previous literature measuring reading skills, our study results indicate that the language used in AI responses generally requires advanced reading skills, typically at a college level. The success of each instrument depends on the literacy and demands of the audience, thus the comprehensive responsiveness alone might not be sufficient from the standpoint of functional literacy.

PEMAT is a validated measurement tool developed to evaluate patient education materials regarding understandability and actionability [28]. Understandability refers to how easy it is for people from different backgrounds and with different degrees of health literacy to read, process, and explain the core message of the materials. Actionability refers to how easy it is for people to determine the steps they need based on the information presented to them.

Looking at the literature, Ayoub et al. analyzed postoperative patient instructions for eight pediatric otolaryngology procedures. They found that instructions created with ChatGPT were scored between 73–82% in terms of understandability and 20–80% in terms of actionability, while instructions created with Google Search were scored between 73–82% in terms of understandability and 40–100% in terms of actionability [49]. As a result of the study, it was reported that ChatGPT’s instructions received lower understandability and actionability scores compared to Google Search. In a study based on ophthalmology patients who underwent surgery, in which this study was taken as a model, it was observed that postoperative instructions created with ChatGPT scored 77% in terms of understandability and 60% in terms of actionability, while instructions created with Google Search scored 69–85% in terms of understandability and 60% in terms of actionability. No difference was found between both groups in terms of understandability and actionability [50]. In a study comparing ChatGPT and Google Bard responses in response to frequently asked questions by patients with obstructive sleep apnea syndrome, the results of PEMAT-P scores for both understandability and actionability were reported to be higher for ChatGPT in all question categories (understandability: 85–92%, actionability: 60–80%) [51]. In our study, compared with these studies, it was observed that the understandability and actionability scores according to PEMAT-P for MC presented values close to the literature. However, both understandability and actionability scores for GS were considerably lower than the literature data. We think that the reason for this difference is that the diagnosis and/or treatment of infertility is a disease that cannot be explained without using short and medical language. During the scoring, we realized how the GS presents the answers may cause limitations in answering some queries effectively. Moreover, studies assessing outcomes like post-operative instructions are favorable regarding actionability from a design perspective. In our study, the trends queries we used were mostly not suitable for actionable answers. This situation resulted in lower actionability scores.

This study demonstrated that both GS and MC tend to deliver answers appropriate for an advanced level of comprehension to the patients seeking answers in the field of infertility. When the understandability and actionability scores are evaluated, MC results provide more comprehensible and more actionable data than GS results. So, MC may be more beneficial when querying information about “Infertility”. We observed that GC provide more brief and spot information, whereas MC most often present detailed infromation and offers remarks from different angles. However, when using such AI-supported LLMs, it should be known that the information provided may not be accurate or misleading and may not be a substitute for actual medical knowledge. AI-supported engines can perform at different levels in each version, and even if new top models are constantly emerging, the reliability of these robots, which draw data from the internet environment where there is much misinformation for knowledge generation, should be questioned.

Our study has some limitations due to the online sources used. In order to determine the queries to be used in the study, a sample of international sources was created at the beginning of the study. For this reason, nine developed countries were selected, and the most frequently searched questions were determined by trends analysis. However, these analyses may need to be repeated for each country due to the different levels of English education in each country or the assumption that many users will query in their native language. Also, more diversity can be provided by using other search engines and AI chatbots, and the superiority of different LLM robots can be analyzed. We also measured the readability, understandability, and actionability of the GS and MC. The information’s quality, relevance, validity, and timeliness are more complex parameters that cannot be assessed by validated tools such as PEMAT. The development of validated scales that analyze this data would add depth to such studies.

Regarding limitations, one limitation that is missing is the number of expert reviewers. Only two reviewers were used, and neither was a native English speaker.

## Conclusion

GS and MC are both pragmatic tools to access medical information with high readibility scores. In terms of presenting medical information to infertility patients, MC provide more understandable and actionable information than GS. Due to their advanced technological infrastructure and development potential, AI chatbots pledge to provide comprehensive information with great speed. However, the reliability of information they provide has not been verified yet. Until further studies on AI tools with higher level of evidence are conducted, clinicians must remain patients’ primary true source of information.

## Supplementary information

Supplementary file 1

## Data Availability

Data are available from the corresponding author on reasonable request.

## References

[1] Organization WH. WHO manual for the standardized investigation and diagnosis of the infertile couple. Cambridge: Cambridge University Press; 2000.

[2] Organization WH. Infertility prevalence estimates, 1990–2021. Geneva: World Health Organization; 2023.

[3] Weissman A, Gotlieb L, Ward S, Greenblatt E, Casper RF. Use of the internet by infertile couples. Fertil Steril. 2000;73:1179–82. 10856479 10.1016/s0015-0282(00)00515-x

[4] Google. How search works [Internet]. Google Developers. https://developers.google.com/search/docs/fundamentals/how-search-works. 2025.

[5] Jeannot JG. [Dr. Google. Google search engine for medicine]. Rev Med Suisse. 2008;4:1280–2. 18616211

[6] Checcucci E, Rodler S, Piazza P, Porpiglia F, Cacciamani GE. Transitioning from “Dr. Google” to “Dr. ChatGPT”: the advent of artificial intelligence chatbots. Transl Androl Urol. 2024;13:1067–70. 38983463 10.21037/tau-23-629 PMC11228672

[7] Passby L, Jenko N, Wernham A. Performance of ChatGPT on specialty certificate examination in dermatology multiple-choice questions. Clin Exp Dermatol. 2024;49:722–7. 37264670 10.1093/ced/llad197

[8] Gilson A, Safranek CW, Huang T, Socrates V, Chi L, Taylor RA, et al. How does ChatGPT perform on the United States medical licensing examination (USMLE)? The implications of large language models for medical education and knowledge assessment. JMIR Med Educ. 2023;9:e45312. 36753318 10.2196/45312 PMC9947764

[9] Kung TH, Cheatham M, Medenilla A, Sillos C, De Leon L, Elepaño C, et al. Performance of ChatGPT on USMLE: potential for AI-assisted medical education using large language models. PLOS Digit Health. 2023;2:e0000198. 36812645 10.1371/journal.pdig.0000198 PMC9931230

[10] Schoch J, Schmelz H-U, Strauch A, Borgmann H, Nestler T. Performance of ChatGPT-3.5 and ChatGPT-4 on the European board of urology (EBU) exams: a comparative analysis. World J Urol. 2024;42:445. 39060792 10.1007/s00345-024-05137-4

[11] Tepe M, Emekli E. Assessing the responses of large language models (ChatGPT-4, gemini, and microsoft copilot) to frequently asked questions in breast imaging: a study on readability and accuracy. Cureus. 2024;16:e59960. 38726360 10.7759/cureus.59960 PMC11080394

[12] Behers BJ, Vargas IA, Behers BM, Rosario MA, Wojtas CN, Deevers AC, et al. Assessing the readability of patient education materials on cardiac catheterization from artificial intelligence chatbots: an observational cross-sectional study. Cureus. 2024;16:e63865. 39099896 10.7759/cureus.63865 PMC11297732

[13] Altıntaş E, Ozkent MS, Gül M, Batur AF, Kaynar M, Kılıç Ö, et al. Comparative analysis of artificial intelligence chatbot recommendations for urolithiasis management: A study of EAU guideline compliance. Fr J Urol. 2024;34:102666. 38849035 10.1016/j.fjurol.2024.102666

[14] Beilby K, Hammarberg K. O-089 using ChatGPT to answer patient questions about fertility: the quality of information generated by a deep learning language model. Hum Reprod. 2023;38:dead093:103.

[15] Chervenak J, Lieman H, Blanco-Breindel M, Jindal S. The promise and peril of using a large language model to obtain clinical information: ChatGPT performs strongly as a fertility counseling tool with limitations. Fertil Steril. 2023;120:575–83. 37217092 10.1016/j.fertnstert.2023.05.151

[16] Gokmen O, Gurbuz T, Devranoglu B, Karaman MI. Artificial intelligence and clinical guidance in male reproductive health: ChatGPT4.0’s AUA/ASRM guideline compliance evaluation. Andrology. 2025;13:176–83. 39016301 10.1111/andr.13693

[17] Palmor M, Scott CH, Krieg SA, Amato P, Rubin ES. Evaluating Chatgpt’s competency as a health education resource for transgender people seeking fertility information. Fertil Steril. 2024;122:e379.

[18] Von Elm E, Altman DG, Egger M, Pocock SJ, Gøtzsche PC, Vandenbroucke JP. The strengthening the reporting of observational studies in epidemiology (STROBE) statement: guidelines for reporting observational studies. Lancet. 2007;370:1453–7. 18064739 10.1016/S0140-6736(07)61602-X

[19] Free Text Readability Consensus Calculator. [Internet]. Readability scoring system. 2024. https://readabilityformulas.com/readability-scoring-system.php.

[20] Flesch R. A new readability yardstick. J Appl Psychol. 1948;32:221. 18867058 10.1037/h0057532

[21] Kincaid JP, Fishburne RP Jr, Rogers RL, Chissom BS. Derivation of new readability formulas (Automated Readability Index, Fog Count and Flesch Reading Ease Formula) for Navy enlisted personnel. Memphis (TN): Chief of Naval Technical Training; 1975. Report No.: 8–75.

[22] Gunning R. The Technique Of Clear Writing. New York, NY: McGraw-Hill; 1952.

[23] McLaughlin G. SMOG grading-a new readability formula. J Reading. 1969;12:639–46.

[24] Coleman M, Liau TL. A computer readability formula designed for machine scoring. J Appl Psychol. 1975;60:283.

[25] Senter R. Automated readability index. AMRL TR. 1967;66:1–14. 5302480

[26] Agency for Healthcare Research and Quality (AHRQ). [Internet]. The patient education materials assessment tool (PEMAT). https://www.ahrq.gov/health-literacy/patient-education/pemat-p.html.

[27] Steiner SM, Slavych BK, Zraick RI. Assessment of online patient education material about dysphagia. Dysphagia. 2023;38:990–1000. 36205800 10.1007/s00455-022-10524-3

[28] Shoemaker SJ, Wolf MS, Brach C. Development of the patient education materials assessment tool (PEMAT): a new measure of understandability and actionability for print and audiovisual patient information. Patient Educ Couns. 2014;96:395–403. 24973195 10.1016/j.pec.2014.05.027 PMC5085258

[29] Koo TK, Li MY. A guideline of selecting and reporting intraclass correlation coefficients for reliability research. J Chiropr Med. 2016;15:155–63. 27330520 10.1016/j.jcm.2016.02.012 PMC4913118

[30] Wang L, Wang J, Wang M, Li Y, Liang Y, Xu D. Using internet search engines to obtain medical information: a comparative study. J Med Internet Res. 2012;14:e74. 22672889 10.2196/jmir.1943 PMC3799567

[31] Mintz Y, Brodie R. Introduction to artificial intelligence in medicine. Minim Invasive Ther Allied Technol. 2019;28:73–81. 30810430 10.1080/13645706.2019.1575882

[32] Chen J, Wang Y. Social media use for health purposes: systematic review. J Med Internet Res. 2021;23:e17917. 33978589 10.2196/17917 PMC8156131

[33] Ayers JW, Poliak A, Dredze M, Leas EC, Zhu Z, Kelley JB, et al. Comparing physician and artificial intelligence chatbot responses to patient questions posted to a public social media forum. JAMA Intern Med. 2023;183:589–96. 37115527 10.1001/jamainternmed.2023.1838 PMC10148230

[34] Cacciamani GE, Bassi S, Sebben M, Marcer A, Russo GI, Cocci A, et al. Consulting “Dr. Google” for prostate cancer treatment options: a contemporary worldwide trend analysis. Eur Urol Oncol. 2020;3:481–8. 31375427 10.1016/j.euo.2019.07.002 PMC9235534

[35] McInnes N, Haglund BJ. Readability of online health information: implications for health literacy. Inform Health Soc Care. 2011;36:173–89. 21332302 10.3109/17538157.2010.542529

[36] Albright J, de Guzman C, Acebo P, Paiva D, Faulkner M, Swanson J. Readability of patient education materials: implications for clinical practice. Appl Nurs Res. 1996;9:139–43. 8771859 10.1016/s0897-1897(96)80254-0

[37] Gunning R. The fog index after twenty years. Int J Bus Commun. 1969;6:3–13.

[38] Klare GR. Readability. Handbook of Reading Research. 1984;1:681–744.

[39] Hadden K, Prince LY, Schnaekel A, Couch CG, Stephenson JM, Wyrick TO. Readability of patient education materials in hand surgery and health literacy best practices for improvement. J Hand Surg Am. 2016;41:825–32. 27291416 10.1016/j.jhsa.2016.05.006

[40] Institute of Medicine (US) Committee on Health Literacy; Nielsen-Bohlman L, Panzer AM, Kindig DA, eds. Health Literacy: A Prescription to End Confusion. Washington (DC): National Academies Press (US); 2004. 25009856

[41] Network JPE. How to create effective written patient learning materials. Montreal, QC: Sir Mortimer B Davis Jewish General Hospital; 2008.

[42] IOM (Institute of Medicine). Informed consent and health literacy: Workshop summary. Washington, DC: The National Academies Press; 2015.

[43] U.S. National Library of Medicine. How to write easy-to-read health materials. MedlinePlus; 2017.

[44] Karimi AH, Shah AK, Hecht CJ II, Burkhart RJ, Acuña AJ, Kamath AF. Readability of online patient education materials for total joint arthroplasty: a systematic review. J Arthroplasty. 2023;38:1392–9. 36716898 10.1016/j.arth.2023.01.032

[45] Musheyev D, Pan A, Loeb S, Kabarriti AE. How well do artificial intelligence chatbots respond to the top search queries about urological malignancies? Eur Urol. 2024;85:13–6. 37567827 10.1016/j.eururo.2023.07.004

[46] Burns C, Bakaj A, Berishaj A, Hristidis V, Deak P, Equils O. Use of generative AI for improving health literacy in reproductive health: case study. JMIR Form Res. 2024;8:e59434. 38986153 10.2196/59434 PMC11336497

[47] Robins S, Barr HJ, Idelson R, Lambert S, Zelkowitz P. Online health information regarding male infertility: an evaluation of readability, suitability, and quality. Interact J Med Res. 2016;5:e25. 27769954 10.2196/ijmr.6440 PMC5097174

[48] Merrick H, Wright E, Pacey AA, Eiser C. Finding out about sperm banking: what information is available online for men diagnosed with cancer? Hum Fertil. 2012;15:121–8. 10.3109/14647273.2012.702936 22746362

[49] Ayoub NF, Lee YJ, Grimm D, Balakrishnan K. Comparison between ChatGPT and google search as sources of postoperative patient instructions. JAMA Otolaryngol Head Neck Surg. 2023;149:556–8. 37103921 10.1001/jamaoto.2023.0704 PMC10141286

[50] Nanji K, Yu CW, Wong TY, Sivaprasad S, Steel DH, Wykoff CC, et al. Evaluation of postoperative ophthalmology patient instructions from ChatGPT and google search. Can J Ophthalmol. 2024;59:e69–e71. 37884271 10.1016/j.jcjo.2023.10.001

[51] Cheong RCT, Unadkat S, McNeillis V, Williamson A, Joseph J, Randhawa P, et al. Artificial intelligence chatbots as sources of patient education material for obstructive sleep apnoea: ChatGPT versus google bard. Eur Arch Otorhinolaryngol. 2024;281:985–93. 37917165 10.1007/s00405-023-08319-9

