# Supplementary material for: Comparative analysis of the effectiveness of microsoft copilot artificial intelligence chatbot and google search in answering patient inquiries about infertility: evaluating readability, understandability, and actionability
Source: Int J Impot Res. 2025 Apr 22;37(12):1002–7. doi: 10.1038/s41443-025-01056-z (PMC12700802; doi:10.1038/s41443-025-01056-z)
Supplement: Supplementary file 1 [file 41443_2025_1056_MOESM1_ESM.docx]

**Supplementary file 1. Google Trends analysis results (Exclusion criteria applied, hence some rank numbers are missing)**

| **Rank** | **Keyword** | **Average Monthly Searches** | **Status** |
| --- | --- | --- | --- |
| 1 | Ivfs | 500.000 | included |
| 2 | Infertility | 50.000 | included |
| 3 | Fertility clinic near me | 50.000 | excluded |
| 4 | IVF cost | 50.000 | excluded |
| 5 | Male fertility test | 50.000 | included |
| 6 | Fertility test for women | 50.000 | included |
| 7 | Fertility specialist near me | 50.000 | excluded |
| 8 | IVF clinic near me | 50.000 | excluded |
| 9 | Fertility testing | 50.000 | included |
| … | … | … | … |
| 13 | IVF procedure | 50.000 | included |
| … | … | … | excluded |
| 15 | Conception | 50.000 | included |
| 16 | Check fertility | 50.000 | included |
| 17 | Azoospermia | 50.000 | included |
| … | … | … | … |
| 22 | In vitro fertilisation | 50.000 | included |
| … | … | … | … |
| 47 | Fertility treatment options | 5000 | included |
| 48 | IVF fertility | 5000 | included |
| … | … | … | … |
| 65 | Increase male fertility | 5000 | included |
| 66 | Unexplained infertility | 5000 | included |
| … | … | … | included |
| 82 | Improve male fertility | 5000 | included |
| … | … | … | … |
| 94 | Coenzyme q10 for fertility | 5000 | included |
| … | … | … | included |
| 123 | Cause of infertility in male | 5000 | included |
| 124 | Cause of infertility in females | 5000 | included |
| … | … | … | … |
| 151 | Best male fertility test | 500 | included |
| … | … | … | … |
| 158 | Azoospermia treatment | 500 | included |
| … | … | … | … |
| 715 | Zift infertility | 50 | excluded |

… Missing rank numbers indicate keywords excluded due to exclusion criteria
